# Supplementary material for: CRISPR/Cas9-Mediated Multi-Locus Promoter Engineering in ery Cluster to Improve Erythromycin Production in Saccharopolyspora erythraea
Source: Microorganisms. 2023 Feb 28;11(3):623. doi: 10.3390/microorganisms11030623 (PMC10059589; doi:10.3390/microorganisms11030623)
Supplement: Supplementary file 1 [file microorganisms-11-00623-s001.zip › microorganisms-2201017-supplementary.pdf]

Supplementary Material

CRISPR/Cas9-mediated multi-locus promoter engineering in *ery* cluster to improve erythromycin production in *Saccharopolyspora erythraea*

Xuemei Zhang[1,2,3,4], Yan Wang[2,3,5], Yue Zhang[2,3,4*], Meng Wang[1,2,3,4*]

[1]School of Life Sciences, Division of Life Sciences and Medicine, University of Science and Technology of China, Hefei 230026, China

[2]Tianjin Institute of Industrial Biotechnology, Chinese Academy of Sciences, Tianjin 300308, China

[3]Key Laboratory of Engineering Biology for Low-Carbon Manufacturing, Tianjin Institute of Industrial Biotechnology, Chinese Academy of Sciences, Tianjin 300308, China

[4]Key Laboratory of Systems Microbial Biotechnology, Chinese Academy of Sciences, Tianjin 300308, China

[5]College of Biotechnology, Tianjin University of Science and Technology, Tianjin 300457, China

*Corresponding authors:

Dr. Yue Zhang, E-mail: zhangy@tib.cas.cn；Dr. Meng Wang, E-mail: wangmeng@tib.cas.cn

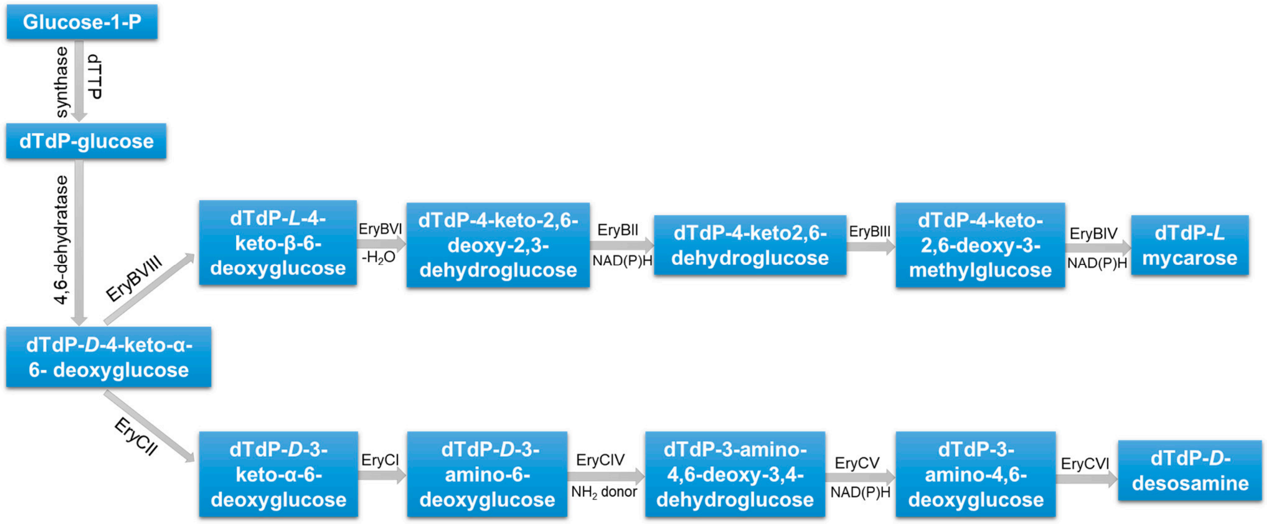

Figure S1. The biosynthetic mechanism of *L*-mycarose and *S*-desosamine.

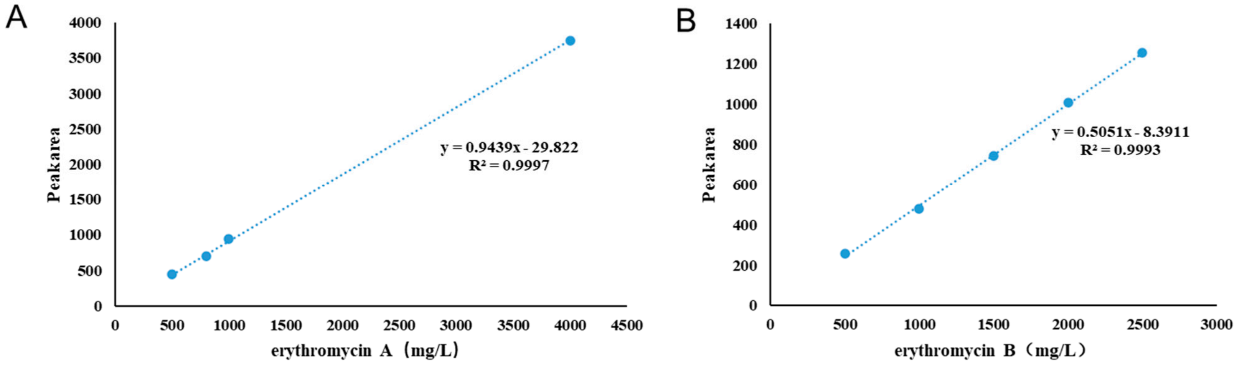

Figure S2. HPLC standard curves of erythromycin A (A) and B (B).

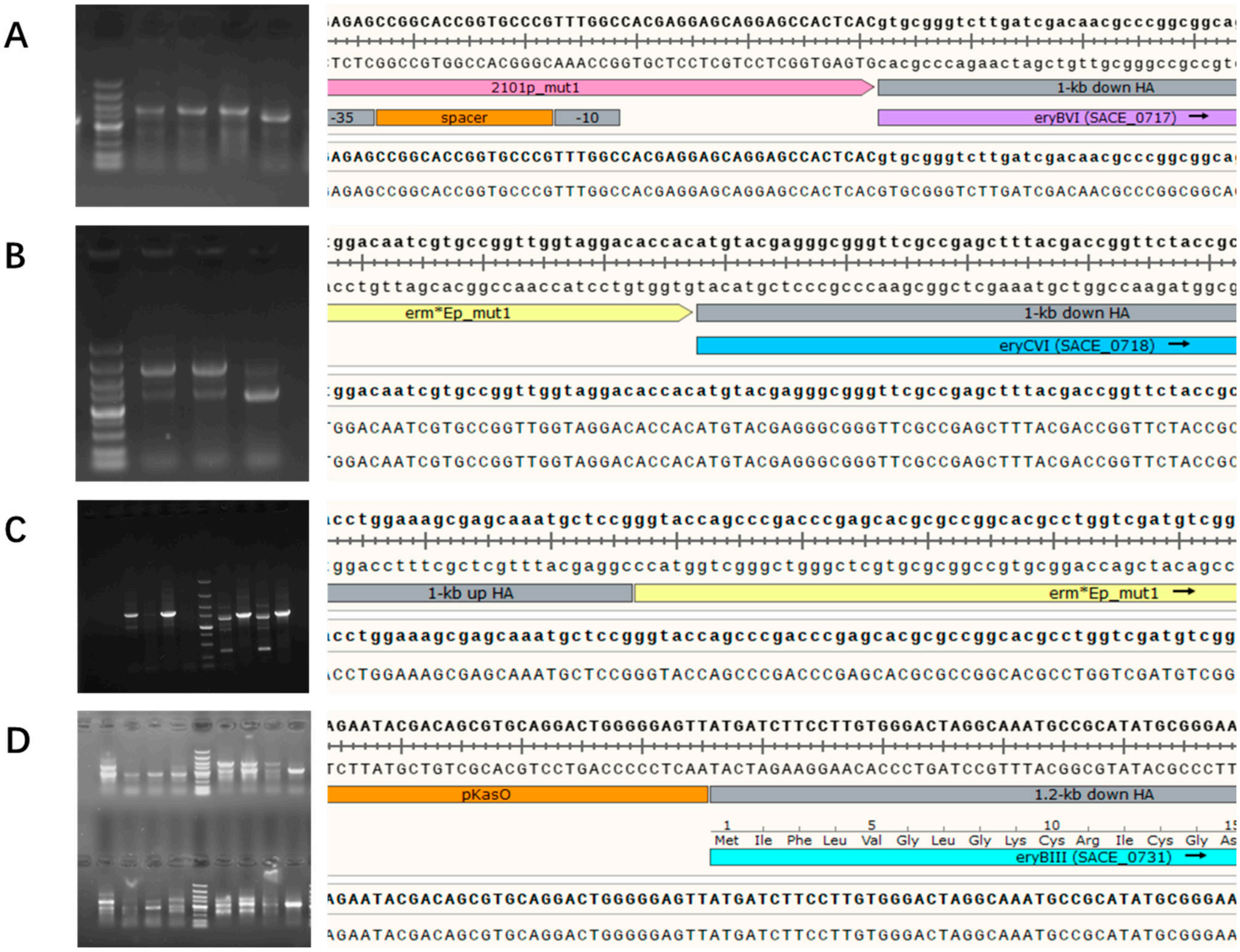

Figure S3. Validation of CRISPR/Cas9 genome editing in L1 (A), L2 (B), L3 (C), and L4 (D) by colony PCR and Sanger sequencing.

Table S1. Strains and plasmids used in this study.

Strain and plasmid	Characteristics	Source
**Strain**		
Saccharopolyspora erythraea NRRL 23338	Wild-type *S. erythraea* strain	Lab stock
Escherichia coli DH5α	Recipient strain used for plasmid construction	Lab stock
Escherichia coli ET12567/pUZ8002	Helper strain used for conjugational transfer harboring plasmid pUZ8002, chloramphenicol and kanamycin resistant	Lab stock
SE/0717(p_{2101_s32})	*S. erythraea* harboring plasmid pCas9-0717(p_{2101_s32})	This study
SE/0717(p_{ermE*_s23})	*S. erythraea* harboring plasmid pCas9-0717(p_{ermE*_s23})	This study
SE/0717(p_{kasO})	*S. erythraea* harboring plasmid pCas9-0717(p_{kasO})	This study
SE/0718(p_{2101_s32})	*S. erythraea* harboring plasmid pCas9-0718(p_{2101_s32})	This study
SE/0718(p_{ermE*_s23})	*S. erythraea* harboring plasmid pCas9-0718(p_{ermE*_s23})	This study
SE/0720(p_{ermE*_s23})	*S. erythraea* harboring plasmid pCas9-0720(p_{ermE*_s23})	This study
SE/0720(p_{kasO})	*S. erythraea* harboring plasmid pCas9-0720(p_{kasO})	This study
SE/0731(p_{2101_s32})	*S. erythraea* harboring plasmid pCas9-0731(p_{2101_s32})	This study
SE/0731(p_{ermE*_s23})	*S. erythraea* harboring plasmid pCas9-0731(p_{ermE*_s23})	This study

SE/0731(p_{kasO})	*S. erythraea* harboring plasmid pCas9-0731(p_{kasO})	This study
*S. erythraea/*pSET152-p_{2101_s32}- egfp(ATG)	*S. erythraea* harboring plasmid pSET152-p_{2101_s32}- egfp(ATG), hygromycin resistant	Lab stock
*S. erythraea/*pSET152-p_{ermE*_s23}- egfp(ATG)	*S. erythraea* harboring plasmid pSET152-p_{ermE*_s23}- egfp(ATG), hygromycin resistant	Lab stock
*S. erythraea/*pSET152-p_{kasO}- egfp(ATG)	*S. erythraea* harboring plasmid pSET152-p_{kasO}- egfp(ATG), hygromycin resistant	This study
*S. erythraea/*pSET152-$p_{ery18\text{-}17}$-egfp(ATG)	*S. erythraea* harboring plasmid pSET152-$p_{ery18\text{-}17}$-egfp(ATG), hygromycin resistant	This study
*S. erythraea/*pSET152-$p_{ery19\text{-}18}$-egfp(ATG)	*S. erythraea* harboring plasmid pSET152-$p_{ery19\text{-}18}$-egfp(ATG), hygromycin resistant	This study
*S. erythraea/*pSET152-$p_{ery21\text{-}20}$-egfp(ATG)	*S. erythraea* harboring plasmid pSET152-$p_{ery21\text{-}20}$-egfp(ATG), hygromycin resistant	This study
*S. erythraea/*pSET152-$p_{ery32\text{-}31}$-egfp(ATG)	*S. erythraea* harboring plasmid pSET152-$p_{ery32\text{-}31}$-egfp(ATG), hygromycin resistant	This study
**Plasmid**		
pCas9-0717(p_{2101_s32})	CRISPR editing plasmid used to replace the *SACE_0717* promoter with p_{2101_s32}, harboring two 1-kb homologous arms (HA), *pSG5* replicon, apramycin resistant	This study
pCas9-0717(p_{ermE*_s23})	CRISPR editing plasmid used to replace the *SACE_0717* promoter with p_{ermE*_s23}, harboring two 1-kb homologous arms (HA), *pSG5* replicon, apramycin resistant	This study

pCas9-0717(p_{kasO})	CRISPR editing plasmid used to replace the *SACE_0717* promoter with p_{kasO}, harboring two 1-kb homologous arms (HA), *pSG5* replicon, apramycin resistant	This study
pCas9-0718(p_{2101_s32})	CRISPR editing plasmid used to replace the *SACE_0718* promoter with p_{2101_s32}, harboring two 1-kb homologous arms (HA), *pSG5* replicon, apramycin resistant	This study
pCas9-0718(p_{ermE*_s23})	CRISPR editing plasmid used to replace the *SACE_0718* promoter with p_{ermE*_s23}, harboring two 1-kb homologous arms (HA), *pSG5* replicon, apramycin resistant	This study
pCas9-0720(p_{ermE*_s23})	CRISPR editing plasmid used to replace the *SACE_0720* promoter with p_{ermE*_s23}, harboring two 1-kb homologous arms (HA), *pSG5* replicon, apramycin resistant	This study
pCas9-0720(p_{kasO})	CRISPR editing plasmid used to replace the *SACE_0720* promoter with p_{kasO}, harboring two 1-kb homologous arms (HA), *pSG5* replicon, apramycin resistant	This study
pCas9-0731(p_{2101_s32})	CRISPR editing plasmid used to replace the *SACE_0731* promoter with p_{2101_s32}, harboring two 1-kb homologous arms (HA), *pSG5* replicon, apramycin resistant	This study
pCas9-0731(p_{ermE*_s23})	CRISPR editing plasmid used to replace the *SACE_0731* promoter with p_{ermE*_s23}, harboring two 1-kb homologous arms (HA), *pSG5* replicon ,apramycin resistant	This study
pCas9-0731(p_{kasO})	CRISPR editing plasmid used to replace the *SACE_0731* promoter with p_{kasO}, harboring two 1-kb homologous arms (HA), *pSG5* replicon, apramycin	This study

	resistant	
pSET152-p_{2101_s32}-egfp(ATG)	Integrated plasmid harboring p_{2101_s32}-*egfp* reporter gene, hygromycin resistant	Lab stock
pSET152-p_{ermE*_s23}-egfp*(*ATG*)*	Integrated plasmid harboring p_{ermE*_s23}-*egfp* reporter gene, hygromycin resistant	Lab stock
pSET152-p_{kasO}- egfp(ATG)	Integrated plasmid harboring p_{kasO}-*egfp* reporter gene, hygromycin resistant	This study
pSET152- pery18-17-egfp(ATG)	Integrated plasmid harboring $p_{ery18-17}$-*egfp* reporter gene, hygromycin resistant	This study
pSET152- pery19-18-egfp(ATG)	Integrated plasmid harboring $p_{ery19-18}$-*egfp* reporter gene, hygromycin resistant	This study
pSET152- pery21-20-egfp(ATG)	Integrated plasmid harboring $p_{ery21-20}$-*egfp* reporter gene, hygromycin resistant	This study
pSET152- pery32-31-egfp(ATG)	Integrated plasmid harboring $p_{ery32-31}$-*egfp* reporter gene, hygromycin resistant	This study

Table S2. Primes used in this study.

Purpose	Primer name	Sequence (5'-3')
**Promoter engineering**		
**For plasmid construction**		
pCas9-0717(p_{2101_s32})	UDGbb-F	GGCCAGGAACCGTAAAAAGG
	pCRIS-1R	CAGACGTAGTCCTTGGTGAGAC TCCGCCCC
	pCRIS-2F	CAGACGTAGTCCTTGGTGAGAC TCCGCCCC
	Cas9(QC)-R	GCGGGGAACGCGTAGATCTG
	ccdB-F2	CAGATCTACGCGTTCCCCGC
	18p17-upHA-R	TCATCCGCGCACACCGACGAA
	18p17-2101p_mut1-F	TTCGTCGGTGTGCGCGGATGAA GTCCGAGTTCGACGCGACCG
	18p17-2101p_mut1-R	TTGTCGATCAAGACCCGCACGT GAGTGGCTCCTGCTCCTC
	18p17-downHA-F	GTGCGGGTCTTGATCGACAACG CC
	18p17-downHA-R	CCTTTTTACGGTTCCTGGCCTTC TTCTCCTCGTGCTCGAT
pCas9-0717(p_{ermE*_s23})	UDGbb-F	GGCCAGGAACCGTAAAAAGG
	pCRIS-1R	CAGACGTAGTCCTTGGTGAGAC TCCGCCCC
	pCRIS-2F	CAGACGTAGTCCTTGGTGAGAC TCCGCCCC

	Cas9(QC)-R	GCGGGGAACGCGTAGATCTG
	ccdB-F2	CAGATCTACGCGTTCCCCGC
	18p17-sgRNA-R	TGACCGGACCCTTACAGTGAGC GTATCCCCTTTCAGATAC
	18p17-sgRNA-F	TCACTGTAAGGGTCCGGTCAGTT TTAGAGCTAGAAATAGC
	ccdB-R2	TCTAGATAAAAAACGCCCGG
	18p17-upHA-F	CCGGGCGTTTTTTATCTAGACCG CAAATATCAGTTGCACA
	18p17-upHA-R	TGCTCGGGTCGGGCTGGTACCTC ATCCGCGCACACCGACGAA
	ermEp_mut1-F	GGTACCAGCCCGACCCGAGCA
	ermEp_mut1-R	GTGGTGTCCTACCAACCGGC
	18p17-downHA-F	GCCGGTTGGTAGGACACCACGT GCGGGTCTTGATCGACAA
	18p17-downHA-R	CCTTTTTACGGTTCCTGGCCTTC TTCTCCTCGTGCTCGAT
pCas9-0717(p_{kasO})	UDGbb-F	GGCCAGGAACCGTAAAAAGG
	pCRIS-1R	CAGACGTAGTCCTTGGTGAGAC TCCGCCCC
	pCRIS-2F	CAGACGTAGTCCTTGGTGAGAC TCCGCCCC
	Cas9(QC)-R	GCGGGGAACGCGTAGATCTG
	ccdB-F2	CAGATCTACGCGTTCCCCGC
	18p17-upHA-R	TCATCCGCGCACACCGACGAA

	18p17-KasOp_mut1-F	TTCGTCGGTGTGCGCGGATGATG TTCACATTCGAACGGTCTCTG
	18p17-KasOp_mut1-R	TTCGTCGGTGTGCGCGGATGATG TTCACATTCGAACGGTCTCTG
	18p17-downHA-F	GTGCGGGTCTTGATCGACAACG CC
	18p17-downHA-R	CCTTTTTACGGTTCCTGGCCTTC TTCTCCTCGTGCTCGAT
pCas9-0718(p_{2101_s32})	UDGbb-F	GGCCAGGAACCGTAAAAAGG
	pCRIS-1R	CAGACGTAGTCCTTGGTGAGAC TCCGCCCC
	pCRIS-2F	CAGACGTAGTCCTTGGTGAGAC TCCGCCCC
	Cas9(QC)-R	GCGGGGAACGCGTAGATCTG
	ccdB-F2	CAGATCTACGCGTTCCCCGC
	19p18-sgRNA-R	ACCGACAAGTCCGTCCGACAGC GTATCCCCTTTCAGATAC
	19p18-sgRNA-F	TGTCGGACGGACTTGTCGGTGTT TTAGAGCTAGAAATAGC
	ccdB-R2	TCTAGATAAAAAACGCCCGG
	19p18-upHA-F	CCGGGCGTTTTTATCTAGACTA CTCCTTCTACCTGGACTTCTA
	19p18-upHA-R	GGTCGCGTCGAACTCGGACTCT AGCCGGCGTGGCGGCGCGTGAG TTCCTCCA
	2101p_mut1-F	AGTCCGAGTTCGACGCGACC
	2101p_mut1-R	GTGAGTGGCTCCTGCTCCTC

	19p18-downHA-F	GAGGAGCAGGAGCCACTCACATGTACGAGGGCGGGTTCGCCGAGCTTT
	19p18-downHA-R	CCTTTTTACGGTTCCTGGCCTGGAACGACCAGCGGTCCAT
pCas9-0718(p_{ermE*_s23})	UDGbb-F	GGCCAGGAACCGTAAAAAGG
	pCRIS-1R	CAGACGTAGTCCTTGGTGAGACTCCGCCCC
	pCRIS-2F	CAGACGTAGTCCTTGGTGAGACTCCGCCCC
	Cas9(QC)-R	GCGGGGAACGCGTAGATCTG
	ccdB-F2	CAGATCTACGCGTTCCCCGC
	19p18-upHA-R	CTAGCCGGCGTGGCGGCGCGTGAGTTCCTCCA
	19p18- ermEp _mut1-F	TGGAGGAACTCACGCGCCGCCACGCCGGCTAGGGTACCAGCCCGACCCGAGCA
	19p18- ermEp _mut1-R	AAAGCTCGGCGAACCCGCCCTCGTACATGTGGTGTCCTACCAACCGGC
	19p18-downHA-F	ATGTACGAGGGCGGGTTCGCCGAGCTTT
	19p18-downHA-R	CCTTTTTACGGTTCCTGGCCTGGAACGACCAGCGGTCCAT
pCas9-0720(p_{ermE*_s23})	UDGbb-F	GGCCAGGAACCGTAAAAAGG
	pCRIS-1R	CAGACGTAGTCCTTGGTGAGACTCCGCCCC
	pCRIS-2F	CAGACGTAGTCCTTGGTGAGACTCCGCCCC

	Cas9(QC)-R	GCGGGGAACGCGTAGATCTG
	ccdB-F2	CAGATCTACGCGTTCCCCGC
	21p20-upHA-R	CGGAGCATTTGCTCGCTTTC
	21p20- ermEp _mut1-F	GAAAGCGAGCAAATGCTCCGGG TACCAGCCCGACCCGAGCA
	21p20- ermEp _mut1-R	CGGGGAATCACTGATCCCATTC ACGTGGTGTCCTACCAACCGGC
	21p20-downHA-F	GTGAATGGGATCAGTGATTCCC CG
	21p20-downHA-R	CCTTTTTACGGTTCCTGGCCACC AGTCCCTGGAAGTGCGT
pCas9-0720(p_{kasO})	UDGbb-F	GGCCAGGAACCGTAAAAAGG
	pCRIS-1R	CAGACGTAGTCCTTGGTGAGAC TCCGCCCC
	pCRIS-2F	CAGACGTAGTCCTTGGTGAGAC TCCGCCCC
	Cas9(QC)-R	GCGGGGAACGCGTAGATCTG
	ccdB-F2	CAGATCTACGCGTTCCCCGC
	21p20-upHA-R	CGGAGCATTTGCTCGCTTTC
	21p20-KasOp_mut1-F	GAAAGCGAGCAAATGCTCCGTGTT CACATTCGAACGGTCTCTGC
	21p20-KasOp_mut1-R	CGGGGAATCACTGATCCCATTCAC AACTCCCCCAGTCCTGCAC
	21p20-downHA-F	GTGAATGGGATCAGTGATTCCC CG
	21p20-downHA-R	CCTTTTTACGGTTCCTGGCCACC AGTCCCTGGAAGTGCGT

pCas9-0731(p_{2101_s32})	UDGbb-F	GGCCAGGAACCGTAAAAAGG
	pCRIS-1R	CAGACGTAGTCCTTGGTGAGACTCCGCCCC
	pCRIS-2F	CAGACGTAGTCCTTGGTGAGACTCCGCCCC
	Cas9(QC)-R	GCGGGGAACGCGTAGATCTG
	ccdB-F2	CAGATCTACGCGTTCCCCGC
	32p31-upHA-R	CACAAGGAAGATCATAACGAGCGC
	32p31- 2101p _mut1-F	GCGCTCGTTATGATCTTCCTTGTGAGTCCGAGTTCGACGCGACCG
	32p31- 2101p _mut1-R	GCCTAGTCCCACAAGGAAGATCATGTGAGTGGCTCCTGCTCCTC
	32p31-downHA-F	ATGATCTTCCTTGTGGGACTAGGC
	32p31-downHA-R	CCTTTTTACGGTTCCTGGCCTCATACGACTTCCAGTCGGG
pCas9-0731(p_{ermE*_s23})	UDGbb-F	GGCCAGGAACCGTAAAAAGG
	pCRIS-1R	CAGACGTAGTCCTTGGTGAGACTCCGCCCC
	pCRIS-2F	CAGACGTAGTCCTTGGTGAGACTCCGCCCC
	Cas9(QC)-R	GCGGGGAACGCGTAGATCTG
	ccdB-F2	CAGATCTACGCGTTCCCCGC
	32p31-sgRNA-R	CATATGCGGCATTTGCCTAGGCGTATCCCCTTTCAGATAC

	32p31-sgRNA-F	CTAGGCAAATGCCGCATATGGT TTTAGAGCTAGAAATAGC
	ccdB-R2	TCTAGATAAAAAACGCCCGG
	32p31-upHA-F	CCGGGCGTTTTTATCTAGATCC CGGATGGCCTTCTTCAG
	32p31-upHA-R	TGCTCGGGTCGGCTGGTACCC ACAAGGAAGATCATAACGA
	ermEp_mut1-F	GGTACCAGCCCGACCCGAGCA
	ermEp_mut1-R	GTGGTGTCCTACCAACCGGC
	32p31-downHA-F	GCCGGTTGGTAGGACACCACAT GATCTTCCTTGTGGGACT
	32p31-downHA-R	CCTTTTTACGGTTCCTGGCCTCA TACGACTTCCAGTCGGG
pCas9-0731(p_{kasO})	UDGbb-F	GGCCAGGAACCGTAAAAAGG
	pCRIS-1R	CAGACGTAGTCCTTGGTGAGAC TCCGCCCC
	pCRIS-2F	CAGACGTAGTCCTTGGTGAGAC TCCGCCCC
	Cas9(QC)-R	GCGGGGAACGCGTAGATCTG
	ccdB-F2	CAGATCTACGCGTTCCCCGC
	32p31-upHA-R	CACAAGGAAGATCATAACGAGC GC
	32p31-KasOp_mut1-F	CGCTCGTTATGATCTTCCTTGTG TGTTCACATTCGAACGGTCTCTG C
	32p31-KasOp_mut1-R	GCCTAGTCCCACAAGGAAGATC ATAACTCCCCCAGTCCTGCAC

	32p31-downHA-F	ATGATCTTCCTTGTGGGACTAGG C
	32p31-downHA-R	CCTTTTTACGGTTCCTGGCCTCA TACGACTTCCAGTCGGG
pSET152-p_{kasO}-egfp(ATG)	pSET152-F	CGCGGCCGCGCGCGATATCG
	pSET152-R	CAGCCCAAGCTTGGCACTGG
	pkasO-egfp(ATG)-F2	CCAGTGCCAAGCTTGGGCTGTGT TCACATTCGAACGGTCTCTGCTT TGACAACATGCTG
	egfp-R	CGATATCGCGCGCGGCCGCGTT ACTTGTACAGCTCGTCCA
pSET152- $p_{ery18\text{-}17}$-egfp(ATG)	pSET152-F	CGCGGCCGCGCGCGATATCG
	pSET152-R	CAGCCCAAGCTTGGCACTGG
	egfp-F	ATGGTCAGCAAGGGCGAGG
	egfp-R	CGATATCGCGCGCGGCCGCGTT ACTTGTACAGCTCGTCCA
	ery 18p17-F	CCAGTGCCAAGCTTGGGCTGCC CGTGCGTTCGCGTTTTCC
	ery 18p17-R	CCTCGCCCTTGCTGACCATTCAC TGTAAGGGTCCGGTCAC
pSET152- $p_{ery19\text{-}18}$-egfp(ATG)	pSET152-F	CGCGGCCGCGCGCGATATCG
	pSET152-R	CAGCCCAAGCTTGGCACTGG
	egfp-F	ATGGTCAGCAAGGGCGAGG
	egfp-R	CGATATCGCGCGCGGCCGCGTT ACTTGTACAGCTCGTCCA

	ery 19p18-F	CCAGTGCCAAGCTTGGGCTGCGGTTTCCGACCGACAAGTC
	ery 19p18-R	CCTCGCCCTTGCTGACCATCCCTGCTCCCTCCGGAGGTG
pSET152- $p_{ery21-20}$-egfp(ATG)	pSET152-F	CGCGGCCGCGCGATATCG
	pSET152-R	CAGCCCAAGCTTGGCACTGG
	egfp-F	ATGGTCAGCAAGGGCGAGG
	egfp-R	CGATATCGCGCGCGGCCGCGTTACTTGTACAGCTCGTCCA
	ery 21p20-F	CCAGTGCCAAGCTTGGGCTGGCGTCCCCCTACTCGACGAC
	ery 21p20-R	CCTCGCCCTTGCTGACCATCGGAGCATTTGCTCGCTTTC
pSET152- $p_{ery32-31}$-egfp(ATG)	egfp-F	ATGGTCAGCAAGGGCGAGG
	pSET152-R	CAGCCCAAGCTTGGCACTGG
	ery 32p31-F	CCAGTGCCAAGCTTGGGCTGGCAAACAATTTTCAGCTTGATCAAC
**For transformant verification**	ery 32p31-R	CCTCGCCCTTGCTGACCATAACGAGCGCGGCGCCGCGG
	2101p_mut1-F	CCGAGTTCGACGCGACCGTG
	2101p_mut1-R	GTGAGTGGCTCCTGCTCCTC
	ermEp_mut1-F	GGTACCAGCCCGACCCGAGCA
	ermEp_mut1-R	GTGGTGTCCTACCAACCGGC
	KasOp_mut1-F	TGTTCACATTCGAACGGTCTCTGC
	KasOp_mut1-R	

		AACTCCCCCAGTCCTGCAC
	18p17-QCYZ-F	GAATGGAAACCGTTGTCGCGC
	18P17-QCYZ-R	GATGTCCTTGACCAGCAGGGC
	19p18-QCYZ-F	CACTTCCAGGGACTGGTCCC
	19P18-QCYZ-R	GATGATGGGCTGGATCCAGTCC
	21p20-QCYZ-F	CTCGCTTCGACGAACGTGCC
	21p20-QCYZ-R	ACTTCCGGGACGATGTCGAAC
	32p31-QCYZ-F	TTGGCGAAGTCGGGCTCGA
	32p31-QCYZ-R	CTTCTTCTTCGGGTCGCTGC
**qRT-PCR**		
Gene-SACE_0716	16RT-F	ATTCCTGCACACCCTCTACGT
	16RT-R	TCGAACTCGCGCACCAGTG
Gene-SACE_0717	17RT-F	TCGACGACACCGCAGGGAGA
	17RT-R	GTCAGCCAGTCGTGGGTTTCC
Gene-SACE_0718	18RT-F	TCGCCGAGCTTTACGACCG
	18RT-R	CAGCCCGGTCACGTCGTC
Gene-SACE_0719	19RT-F	ACTTCCAGGGACTGGTCCCG
	19RT-F	ACTTCCGGGACGATGTCGAACA
Gene-SACE_0720	20RT-F1	CGCGTCAATTGATCACCCTT
	20RT-R1	ACCAGGTGCACGATCACGTC
Gene-SACE_0731	31RT-F	CAAATGCCGCATATGCGGGA
	31RT-R	GCGCACTTGACCAGTTCGAG

Reference gene	sigA (1801)-F	TCTTGGCCGCAGAACTCTTG
	sigA (1801)-R	TCCAGCTCCGCTGCAAACTC

Table S3. Sequences of the promoters used in this study.

Gene number	Sequence (5'-3')	Length(bp)
pery18-17	CCCGTGCGTTCGCGTTTTCCGTTCCTGGCACAGG<br>TGATCCGCTCCACGGGCCCTTTCCCCGCCGTGAC<br>CGGACCCTTACAGTGA	84
pery19-18	CGGTTTCCGACCGACAAGTCCGTCCGACAGCACA<br>CCTCCGGAGGGAGCAGGG	52
pery21-20	GCGTCCCCCTACTCGACGACCACGCAATGGGCG<br>AACAATATAGGAAGGATCAAGAGGTTGACATCG<br>CCTCGTCGAGCCAACGAACCTGTGAACATCTGCA<br>TGTTGACAAGATCAACGGCGGCTACCTACTGTGG<br>TGGCCCAGTGACGGGTTGCCGCACATCGCGCTGG<br>GGAGATTCTTTGAATTTCGCCCGTAGCACCGACC<br>TGGAAAGCGAGCAAATGCTCCG	224
pery32-31	GCAAACAATTTTCAGCTTGATCAACCTAACACCG<br>GCGCGCGGTGCCGACACGGACTTTTCGGACGCC<br>GCTCCTCGGCCGGACATCGCAGCGGAAAGCGCG<br>ATCCACAATGGACGCAACGGGAGTCGTAAACCG<br>GCTGATGTGATCCAGGACACAAAGCTCGACGGG<br>TTGGCCGCGGCGCCGCGCTCGTT	189
p2101_s32	AGTCCGAGTTCGACGCGACCGTGGTCAACGCCG<br>ACGTGCGGTCGGCGACCAGCGAATTGATAGGAT<br>TGGTGGTCGGCCGCCAACGGTGAGACCGTTCGA<br>CGCGGACCGGGGCGACCCGGTTCGCGCCGACGC<br>GCAGCCGGACGGGGGCGGTCGGACGGCTGCCGC<br>CGAGCGGACCACGCCCAGCGAGCCCGGCCCGCC<br>AGGACCGGCCCCGGCCGGGCCTGGAGCCGGTCG<br>GCACTGCCGGACGCCCGCCGCGCCCGTCGGCGC<br>ACAAGCCGAGAGCCGGCACCGGTGCCCGTTTGG<br>CCACGAGGAGCAGGAGCCACTCAC	321
*permE*_s23*	GGTACCAGCCCGACCCGAGCACGCGCCGGCACG<br>CCTGGTCGATGTCGGACCGGAGTTCGAGGTACGC	282

	GGCTTGCAGGTCCAGGAAGGGGACGTCCATGCG<br>AGTGTCCGTTCGAGTGGCGGCTTGCGCCCGATGC<br>TAGTCGCGGTTGATCGGCGATCGCAGGTGCACGC<br>GGTCGATCTTGACGCTCTGGGGTTGTGAAGTAGA<br>GGATCTGACCGACGCGGTCCACACGTGGCACCG<br>CGATGCTGTTGTGGGCTGGACAATCGTGCCGGTT<br>GGTAGGACACCAC	
pkasO	TGTTCACATTCGAACGGTCTCTGCTTTGACAACA<br>TGCTGTGCGGTGTTGTAAAGTCGTGGCCAGGAGA<br>ATACGACAGCGTGCAGGACTGGGGGAGTT	97

Table S4. Sequences of guide RNA (gRNA) used in this study.

Target	gRNA sequence	PAM
pery18-17	TCACTGTAAGGGTCCGGTCA	CGG
pery19-18	TGTCGGACGGACTTGTCGGT	CGG
pery21-20	GCGTCAATTGATCACCCTTC	TGG
pery32-31	CTAGGCAAATGCCGCATATG	CGG
